# G Protein-Coupled Estrogen Receptor: Rapid Effects on Hippocampal-Dependent Spatial Memory and Synaptic Plasticity

**DOI:** 10.3389/fendo.2020.00385

**Published:** 2020-06-10

**Authors:** Ashok Kumar, Thomas C. Foster

**Affiliations:** Department of Neuroscience, McKnight Brain Institute, University of Florida, Gainesville, FL, United States

**Keywords:** estrogen, estrogen receptor, GPER, spatial memory, synaptic plasticity

## Abstract

In the hippocampus, estrogen regulates gene transcription linked to neuronal growth, neuroprotection, and the maintenance of memory function (1–3). The mechanism is likely to involve genomic regulation through classic estrogen receptor (ER) signaling cascades that influence transcription. Estrogens binding to classic nuclear ERs, alpha (ERα) and beta (ERβ), and have pleotropic effects on development, behavior, and neurophysiological functions, including synaptic plasticity and memory consolidation (4–7). In addition to ERα and ERβ, estrogen can also initiate activation of classical second messenger signaling cascades to influence the activity of G-proteins and a host of kinases, resulting in rapid changes in physiology (8–14). These rapid effects of estrogen are commonly mediated by membrane receptors. In the late 90s, multiple laboratories cloned cDNA/gene for an orphan G-protein-coupled receptor with very low homology with other G-protein-coupled receptors and named it G-protein-coupled receptor 30 (GPR30) (15–20). Later in 2007, the International Union of Basic and Clinical Pharmacology designated GPR30 as G protein-coupled estrogen receptor (GPER) (21); GPER is a seven-transmembrane G-protein-coupled receptor, predominantly expressed on the cell membrane (22). Interestingly, GPER is reported to mediate many of the rapid responses of estradiol in the adult brain, and is widely distributed in the mammalian brain including the plasma membrane of hippocampal neurons (23–31). GPER modulates second messenger signaling cascades involving Gα_s_- and Gα_i/o_-associated increase in cyclic adenosine monophosphate and phosphoinositide 3-kinase or Src protein kinase respectively (32, 33). Activation of GPER is also associated with phospholipase C, and the inositol receptor and ryanodine receptor-mediated increase in intracellular calcium (24, 34). This commentary is concentrated specifically on the possible rapid effects of GPER in hippocampal-dependent spatial memory function and synaptic plasticity.

## Role of GPER in Hippocampal-Dependent Spatial Memory

In hippocampal neurons, GPER immunoreactivity is associated with the plasma membrane and endoplasmic reticulum, along with axon terminals and dendritic spines (22, 24, 29–31, 35–40). It is well established that estrogen can influence synaptic function and improve memory (12, 41–49). G1, a nonsteroidal high-affinity selective GPER agonist, does not bind classical ERs (50), but similar to estrogen, improves cognitive performance, including social recognition, spatial working memory, and long-term spatial memory consolidation (51–59). Results from recent studies by the Frick group, elegantly demonstrate that like 17-beta estradiol (E2), activation of GPER, by direct infusion of G1 into the dorsal hippocampus, can facilitate object recognition memory and hippocampal-dependent spatial memory in ovarectimized female mice. The enhancement of memory was not due to activation of the extracellular signal-regulated kinase signaling normally observed following E2 treatment. Rather, GPER activation was associated with phosphorylation of c-Jun N-terminal, cofilin-mediated actin polymerization, and spinogenesis in region CA1 (55, 57). Overall, these studies provide strong evidence that like E2, activation of GPER can facilitate hippocampal-dependent memory performance.

## GPER and Hippocampal Synaptic Function

**Figure 1:**
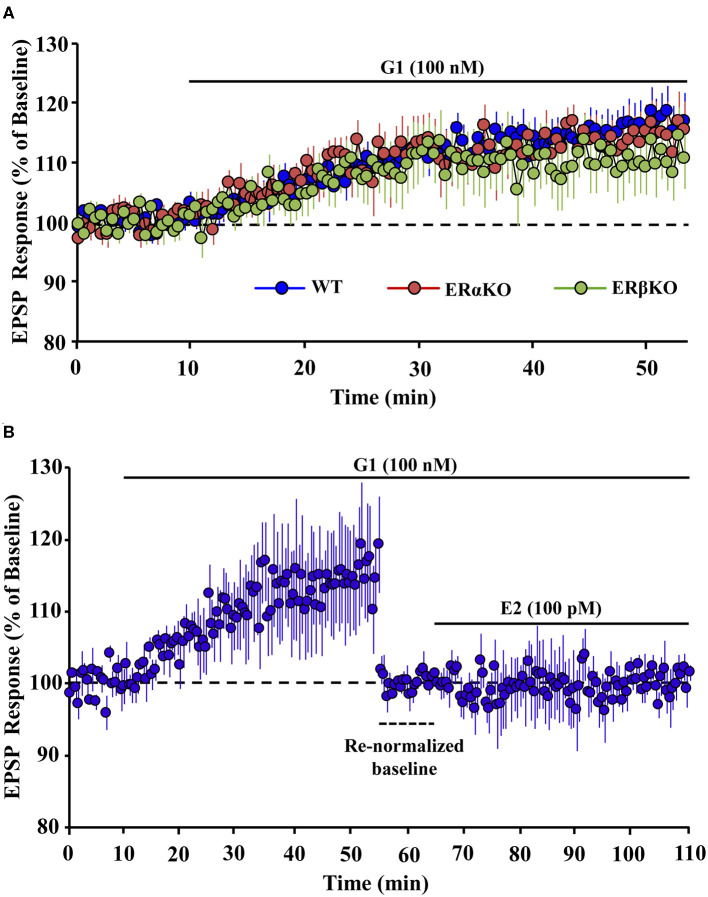
Effect of GPER selective agonist, G1 on hippocampal synaptic responses. **(A)** Time course of the field EPSP measurements on slices obtained from wild type (WT, blue), estrogen receptor (ER) alpha knockout (ERαKO, red), and ER beta knockout (ERβKO, green) mice obtained 10 min before and 45 min after application of G1. **(B)** G1 blocked the 17-beta estradiol (E2)-induced enhanced synaptic responses in hippocampal slices. Time course of field EPSP measurements obtained from hippocampal slices 10 min before and 45 min after G1 application. Bath application of G1 significantly enhanced the synaptic response. Baseline was re-normalized from last 10 min recording (dashed line) following the start of G1 application, and E2 was bath applied in the continued presence of G1. E2 in presence of G1 failed to further enhance synaptic response. Adapted from Kumar et al. (11). Copyright permission granted order # 480097130349.

In addition to enhancing memory performance, GPER activation also contributes to synaptic plasticity. Activation of GPER enhances synaptic transmission at hippocampal CA3-CA1 synapses (11, 54, 60, 61). We recently demonstrated that GPER is a major component of E2-mediated upregulation in extracellular signal-regulated kinase and the rapid facilitation of synaptic responses at CA3-CA1 hippocampal synapses of ovariectomized mice. In addition, the GPER agonist, G1, induced an increase of excitatory postsynaptic potentials (EPSPs) in hippocampal slices obtained from ovariectomized ER alpha knockout (ERαKO) and ER beta knockout (ERβKO) mice (Figure 1A). Confirmation that GPER is a mechanism for rapid E2 effects on synaptic transmission was proven by demonstrating that prior application of G1 blocked the E2-induced enhancement of synaptic responses in hippocampal slices (Figure 1B), while bath application of E2 in absence of G1 increases synaptic responses (11). Interestingly, Oberlander and Woolley demonstrated that GPER-induced potentiation of excitatory synaptic responses in CA1 hippocampal pyramidal neurons is restricted to females and involves postsynaptic mechanisms (61). The role of GPER in synaptic plasticity is still evolving (62–65); however, a number of recent studies indicate that activation of GPER contributes to a rapid increase in hippocampal dendritic spinogenesis and spine density (11, 54, 57, 60, 61, 66, 67).

## Concluding Statement

In many ways, the effects of E2 are opposite to that of aging (3, 68). Recent findings indicate that similar to E2, GPER participates in the rapid effects of the E2-induced increase in hippocampal synaptic transmission and improved cognition. Thus, it will be interesting for future research to explore changes in GPER expression or function over the life span, and their contribution to impaired cognitive and synaptic function associated with aging and neurodegenerative diseases.

## Data Availability Statement

The raw data supporting the conclusions of this article will be made available by the authors, without undue reservation.

## Ethics Statement

The animal study was reviewed and approved by University of Florida.

## Author Contributions

All authors listed have made a substantial, direct and intellectual contribution to the work, and approved it for publication.

## Conflict of Interest

The authors declare that the research was conducted in the absence of any commercial or financial relationships that could be construed as a potential conflict of interest.
